# Supplementary material for: Unveiling Multistability in Urban Traffic Through Percolation Theory and Network Analysis
Source: Entropy (Basel). 2025 Jun 22;27(7):668. doi: 10.3390/e27070668 (PMC12295039; doi:10.3390/e27070668)
Supplement: Supplementary file 1 [file entropy-27-00668-s001.zip › entropy-3692684-supplementary.pdf]

entropy

Article

Supplementary Materials for "Unveiling Multistability in Urban Traffic through Percolation Theory and Network Analysis"

Rui Chen [1], Jiazhen Liu [1,*], Yong Li [1], and Yuming Lin [2,3,*]

[1] Department of Electronic Engineering, Tsinghua University, Beijing 100086, China; akashicr@163.com (R.C.); liyong07@tsinghua.edu.cn (Y.L.)
[2] School of Architecture, Department of Urban Planning and Design, Tsinghua University, Beijing 100086, China
[3] Technology Innovation Center for Smart Human Settlements and Spatial Planning & Governance, Ministry of Natural Resources, Beijing 100812, China
* Correspondence: ljz21135@126.com (J.L.); linyuming9@mail.tsinghua.edu.cn (Y.L.)

1. Symbol Table and Model Parameters

Table S1. Symbolic Representation of Index.

Index	Description
G	Size of the largest connected subgraph in the road network
f	Congestion rate
v	An individual node, denoting a specific road in the traffic network.
s, t	Different nodes, used to represent distinct node pairs in the graph
σ_{st}	The number of shortest paths from node s to node t in the network.
$\sigma_{st}(v)$	The number of shortest paths from node s to node t that pass through node v.
$B(v)$	The betweenness centrality of the road v, measuring the importance of the node in terms of connecting other nodes along the shortest paths.

Table S2. XGBoost Model Parameters.

Parameter	Value
`n_estimators`	100
`max_depth`	6
`learning_rate`	0.1
`subsample`	1.0
`colsample_bytree`	1.0
`random_state`	44
`reg_alpha`	0.1
`reg_lambda`	0.1

Academic Editor: Antonio M. Scarfone

Received: 26 May 2025
Revised: 19 June 2025
Accepted: 20 June 2025
Published: 22 June 2025

Citation: Chen, R.; Liu, J.; Li, Y.; Lin, Y. Unveiling Multistability in Urban Traffic through Percolation Theory and Network Analysis. *Entropy* **2025**, *27*, 668. https://doi.org/10.3390/e27070668

Copyright: © 2025 by the authors. Licensee MDPI, Basel, Switzerland. This article is an open access article distributed under the terms and conditions of the Creative Commons Attribution (CC BY) license (https://creativecommons.org/licenses/by/4.0/).

2. Algorithms

Algorithm 1 Percolation Simulation for Urban Traffic Networks

Input:
List of cities $\mathcal{C}$, date range $[t_{\text{start}}, t_{\text{end}}]$
Road segment geometry files, adjacency matrix files, congestion data files
Number of random runs N_{rand}
for each city $c \in \mathcal{C}$ **do**
Load road network:
Read road segment geometry and IDs for city c
Read adjacency matrix; build directed graph G_c with segments as nodes and adjacency as edges
Remove nodes not present in road segment data
Identify largest connected component of G_c
Load congestion data for city c **in given time window**
for each time snapshot t in the morning peak **do**
Calculate congestion rate f_t = (number of congested roads at t)/(total number of roads)
Identify top f_t fraction of road segments with highest congestion
Remove these segments from G_c to obtain $G'_c(t)$
Compute $G(f_t)$ = size of the largest connected component in $G'_c(t)$
Record $(f_t, G(f_t))$ for empirical percolation curve
end for
for each congestion rate f_r in a predefined range **do**
for $i = 1, \ldots, N_{\text{rand}}$ **do**
Randomly select f_r fraction of road segments
Remove selected segments from G_c to obtain $G_c^{(i)}$
Compute $G_r^{(i)}(f_r)$ = size of the largest connected component in $G_c^{(i)}$
end for
Aggregate $\{G_r^{(i)}(f_r)\}_{i=1}^{N_{\text{rand}}}$ to obtain mean and variance for random removal
end for
if betweenness-based removal is needed **then**
for each congestion rate f_b **do**
Compute (weighted) betweenness centrality for all segments
Remove top f_b fraction of segments by betweenness from G_c
Compute $G_b(f_b)$ = size of largest connected component after removal
Record $(f_b, G_b(f_b))$
end for
end if
Output for city c**:**
Empirical percolation curve $(f_t, G(f_t))$
Random removal curves $(f_r, \langle G_r(f_r) \rangle)$
(Optional) Betweenness-based removal curves $(f_b, G_b(f_b))$
end for

Algorithm 2 Clustering of Stable States in Urban Traffic Networks via DBSCAN

Input:
Empirical percolation data for city c: time series of (G_t, f_t) pairs (normalized largest component size and congestion rate for each time t)
Parameter ranges for DBSCAN: ϵ (radius), minPts (minimum neighbors)
Preprocessing:
Standardize all (G, f) pairs to zero mean and unit variance to ensure isotropic clustering performance
Parameter Selection:
Define candidate grids for ϵ and minPts based on data scale and prior studies
for each parameter combination $(\epsilon, \text{minPts})$ **do**
  Apply DBSCAN to standardized (G, f) pairs
  Compute silhouette score or other clustering validity metric
end for
Select $(\epsilon^*, \text{minPts}^*)$ with maximal mean silhouette score (or best cluster separation)
Clustering:
Apply DBSCAN to standardized data using $(\epsilon^*, \text{minPts}^*)$
Each data point is assigned to a cluster (dense region, interpreted as stable state) or labeled as noise (transitional or outlier state)
Postprocessing and Interpretation:
Map cluster labels back to original data points
Visualize clusters in (G, f) space, color-coded by cluster, for physical interpretation
Identify number and range of stable states for each city; analyze correlation with road network topology
Output:
Cluster assignment for each time snapshot
Number, range and transition pattern of stable states in each city
(Optional) Fraction and distribution of noise points (transitional/unstable periods)

Algorithm 3 Prediction of Global Network State via XGBoost

Input:
Historical congestion data for all road segments in city c
Betweenness centrality values for all segments
Thresholds for selecting top p% critical segments ($p \in \{0.05, 0.1, 0.5, 1\}$)
DBSCAN cluster labels, global congestion rate f, and largest component size G for each snapshot
for each selection threshold p **do**
  Identify set S_p of road segments with top p% betweenness values
  for each time snapshot t **do**
    Construct feature vector $\mathbf{x}_t$ = binary congestion status of segments in S_p
    Collect target variables: DBSCAN state label $y_t^{(1)}$, congestion rate f_t, and component size G_t
  end for
  Data Splitting:
Randomly partition data into training set (70%) and testing set (30%)
  Model Training and Evaluation:
  for each prediction task **do**
    Train XGBoost model on training set
    Optimize hyperparameters via cross-validation
    Evaluate performance on test set:
– Classification: accuracy, AUC, F1-score
– Regression: MSE, MAE, R^2
  end for
  Store results for each p
end for
Output:
Model performance metrics for all selection thresholds p (see Tables 2–4)

3. Supplementary Figures

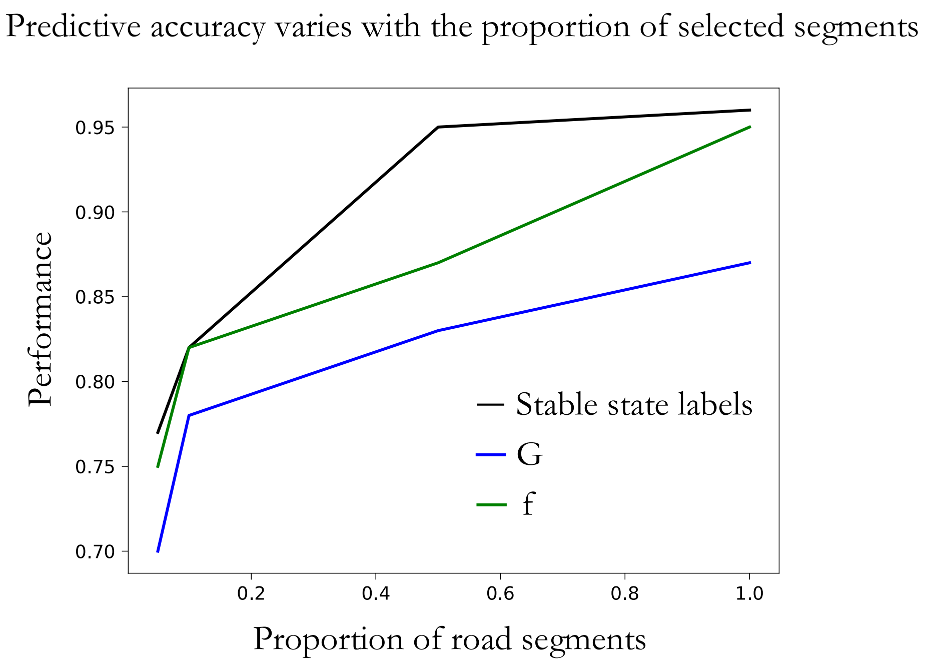

Figure S1. Predictive accuracy varies with the proportion of selected segments.

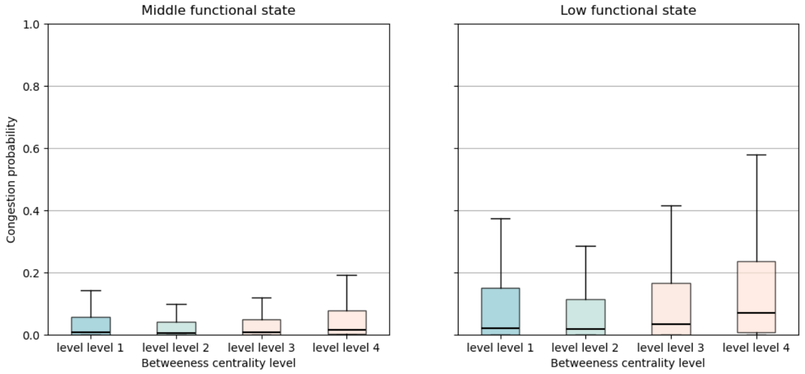

Figure S2. Congestion probability of betweeness centrality levels in Beijing, grouped by functional traffic states.

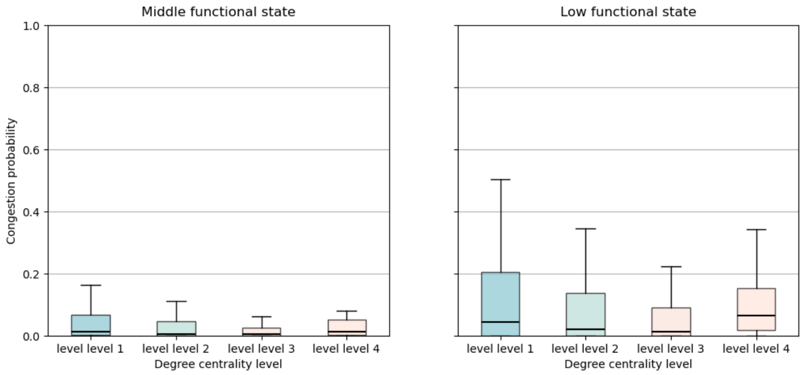

Figure S3. Congestion probability of degree centrality levels in Beijing, grouped by functional traffic states.

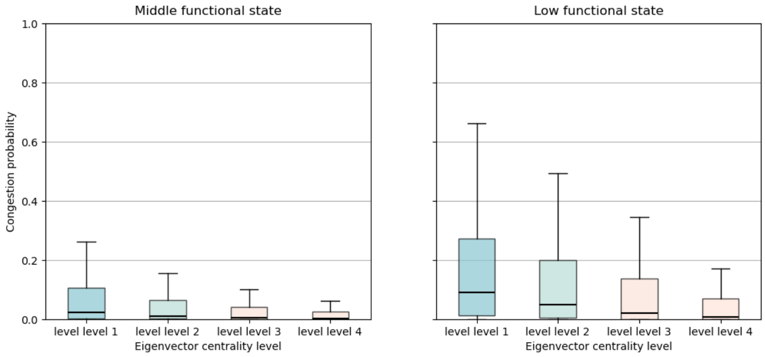

Figure S4. Congestion probability of eigenvector centrality levels in Beijing, grouped by functional traffic states.

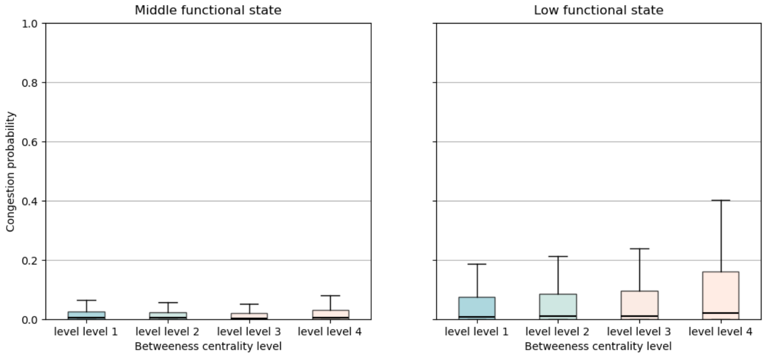

Figure S5. Congestion probability of betweeness centrality levels in Changsha, grouped by functional traffic states.

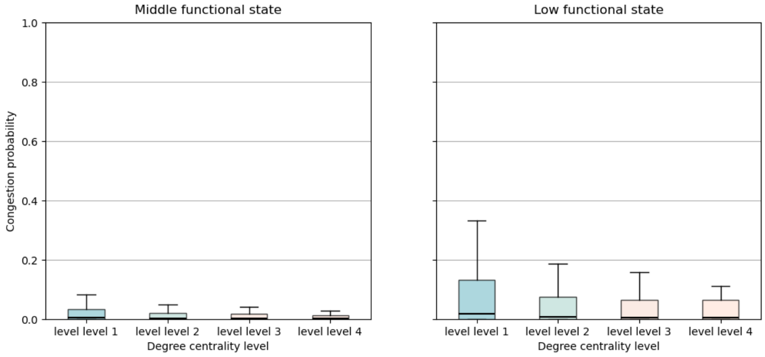

Figure S6. Congestion probability of degree centrality levels in Changsha, grouped by functional traffic states.

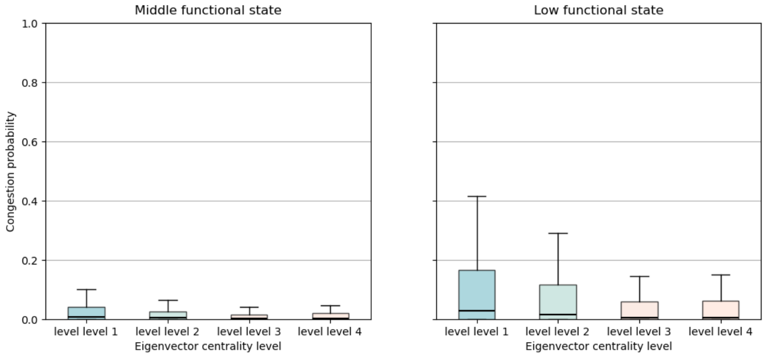

Figure S7. Congestion probability of eigenvector centrality levels in Changsha, grouped by functional traffic states.

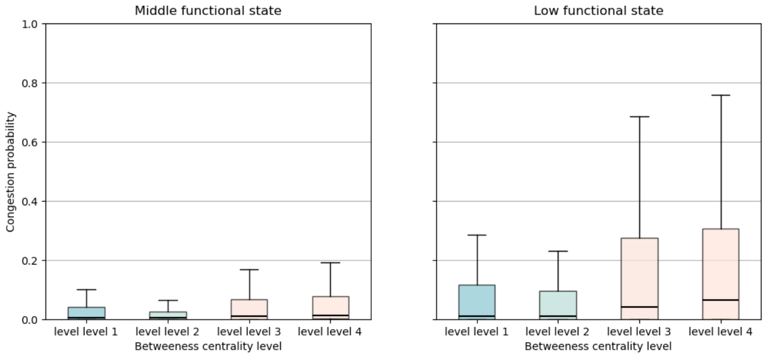

Figure S8. Congestion probability of betweeness centrality levels in Chengdu, grouped by functional traffic states.

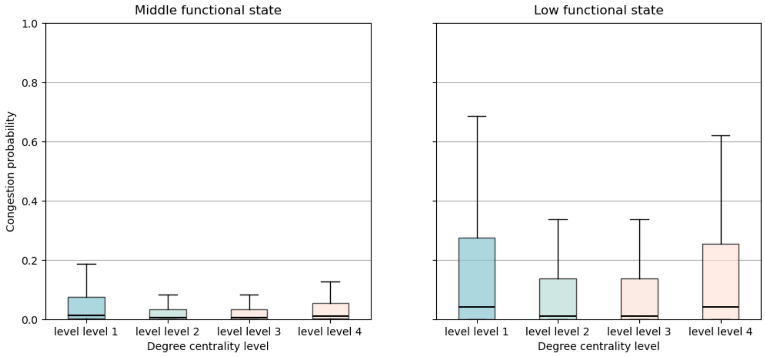

Figure S9. Congestion probability of degree centrality levels in Chengdu, grouped by functional traffic states.

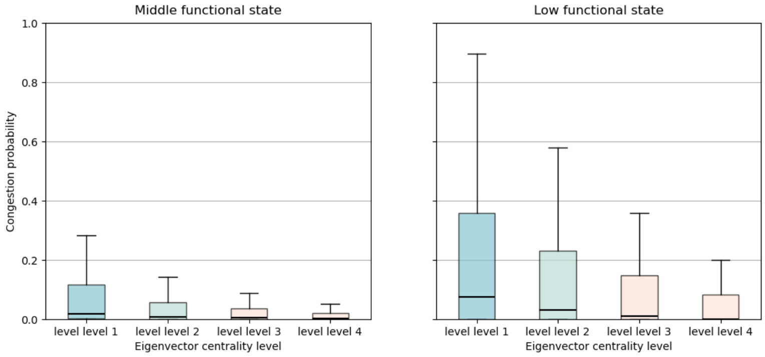

Figure S10. Congestion probability of eigenvector centrality levels in Chengdu, grouped by functional traffic states.

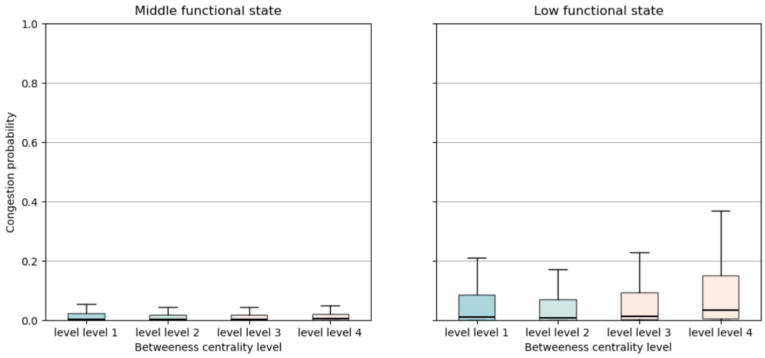

Figure S11. Congestion probability of betweeness centrality levels in Guangzhou, grouped by functional traffic states.

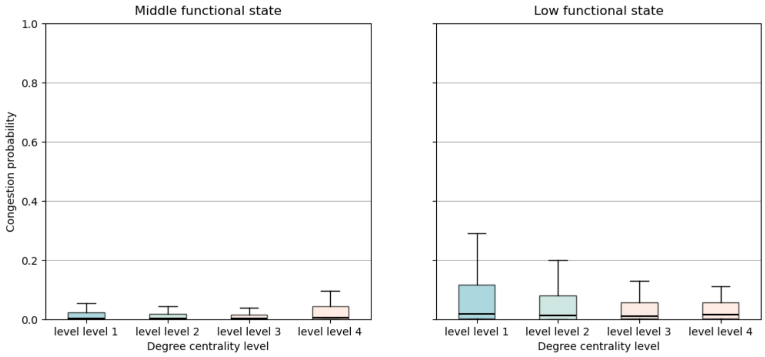

Figure S12. Congestion probability of degree centrality levels in Guangzhou, grouped by functional traffic states.

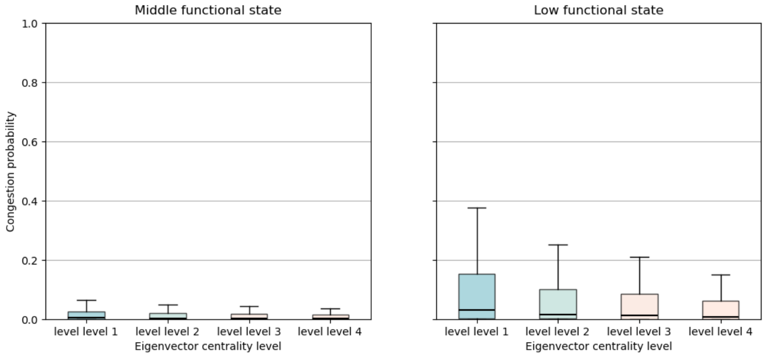

Figure S13. Congestion probability of eigenvector centrality levels in Guangzhou, grouped by functional traffic states.

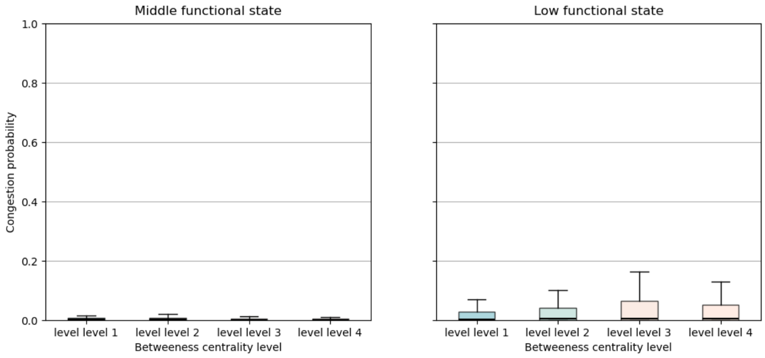

Figure S14. Congestion probability of betweeness centrality levels in Guiyang, grouped by functional traffic states.

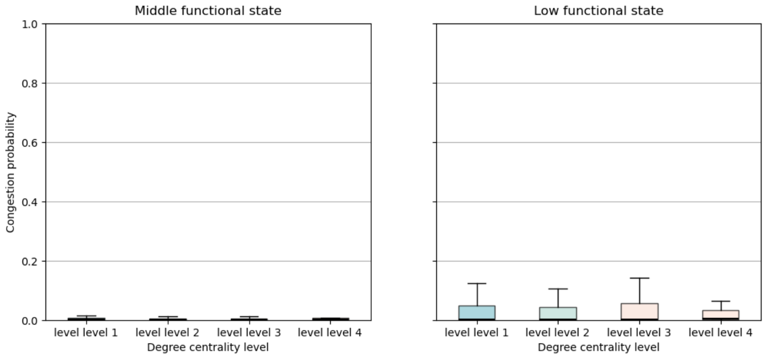

Figure S15. Congestion probability of degree centrality levels in Guiyang, grouped by functional traffic states.

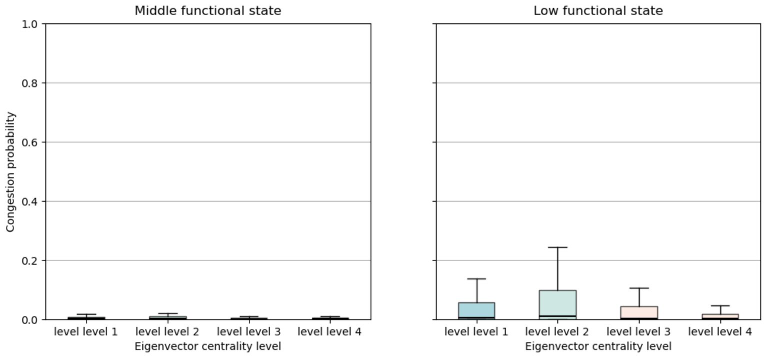

Figure S16. Congestion probability of eigenvector centrality levels in Guiyang, grouped by functional traffic states.

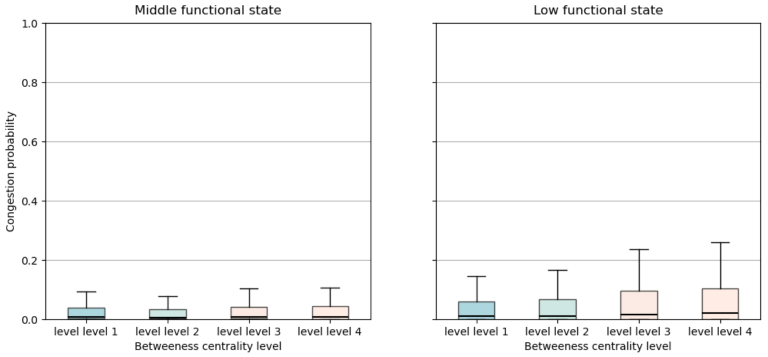

Figure S17. Congestion probability of betweeness centrality levels in Hangzhou, grouped by functional traffic states.

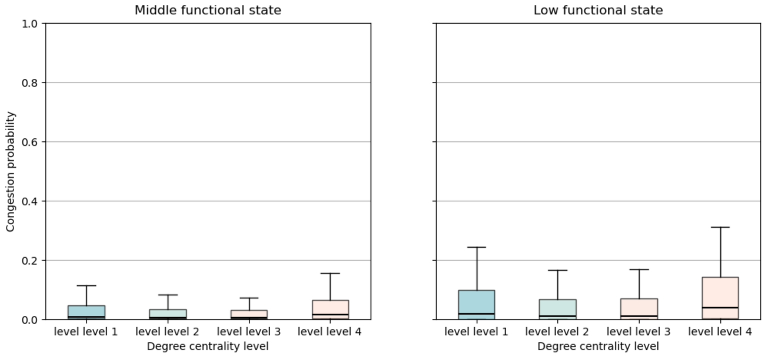

Figure S18. Congestion probability of degree centrality levels in Hangzhou, grouped by functional traffic states.

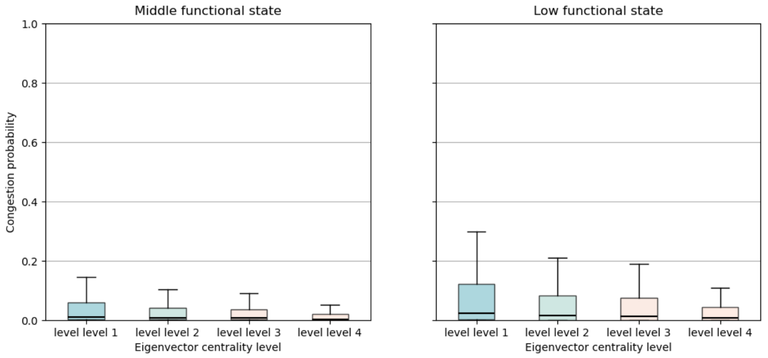

Figure S19. Congestion probability of eigenvector centrality levels in Hangzhou, grouped by functional traffic states.

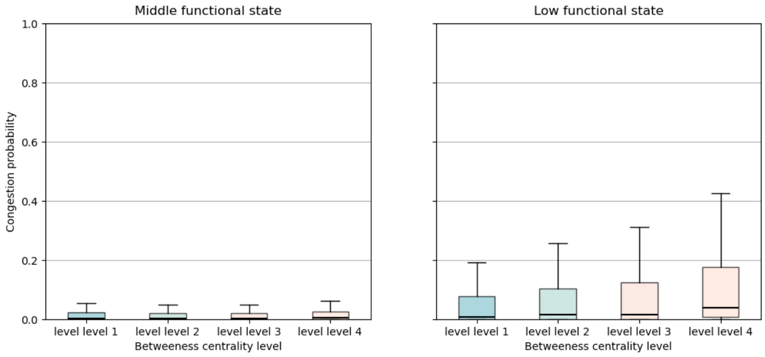

Figure S20. Congestion probability of betweeness centrality levels in Jinan, grouped by functional traffic states.

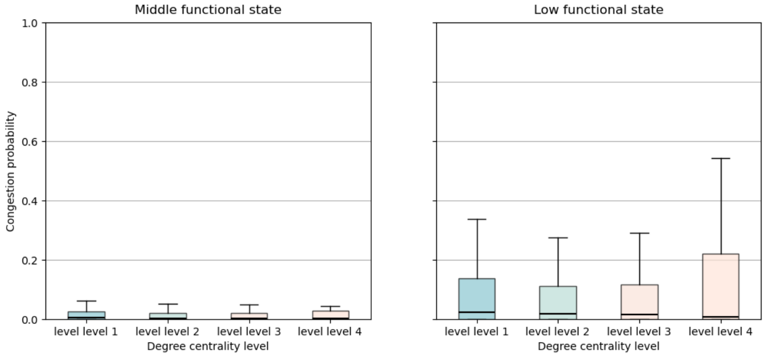

Figure S21. Congestion probability of degree centrality levels in Jinan, grouped by functional traffic states.

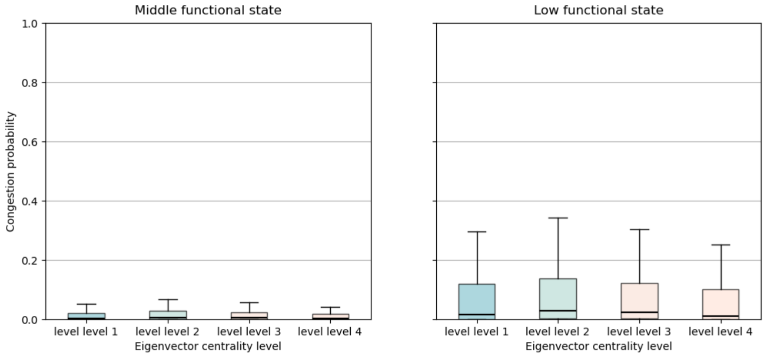

Figure S22. Congestion probability of eigenvector centrality levels in Jinan, grouped by functional traffic states.

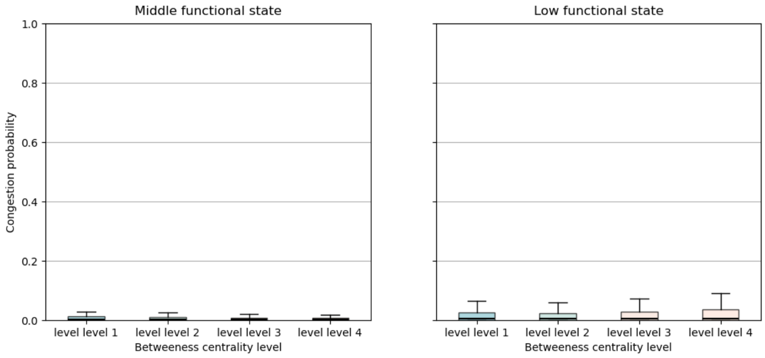

Figure S23. Congestion probability of betweeness centrality levels in Nanjing, grouped by functional traffic states.

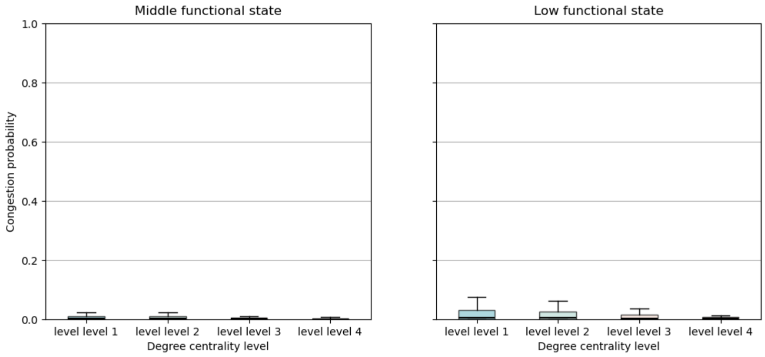

Figure S24. Congestion probability of degree centrality levels in Nanjing, grouped by functional traffic states.

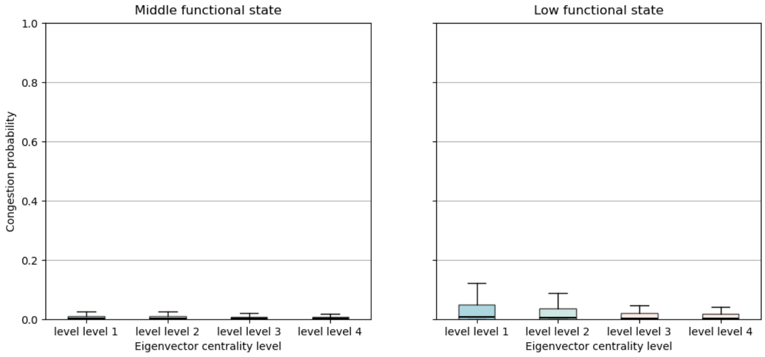

Figure S25. Congestion probability of eigenvector centrality levels in Nanjing, grouped by functional traffic states.

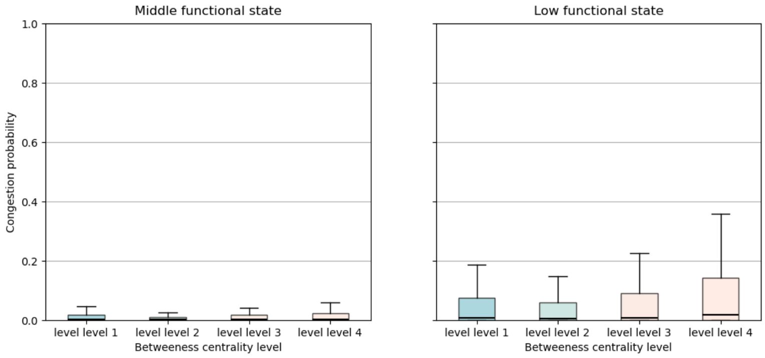

Figure S26. Congestion probability of betweeness centrality levels in Shanghai, grouped by functional traffic states.

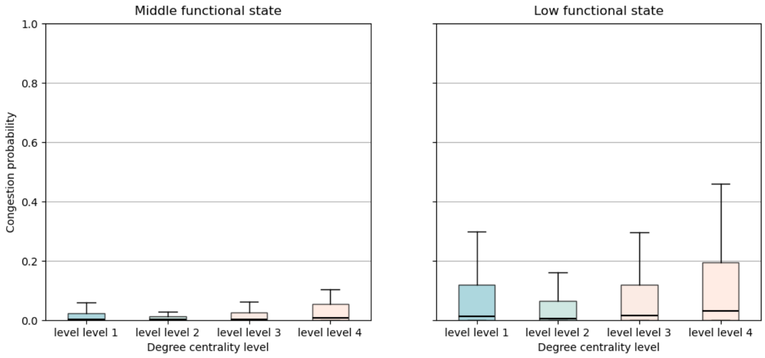

Figure S27. Congestion probability of degree centrality levels in Shanghai, grouped by functional traffic states.

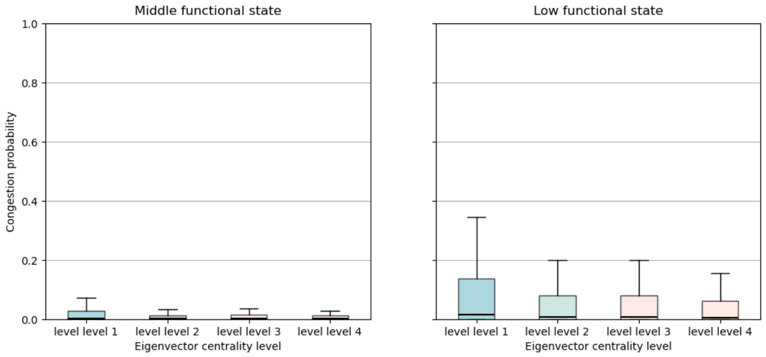

Figure S28. Congestion probability of eigenvector centrality levels in Shanghai, grouped by functional traffic states.

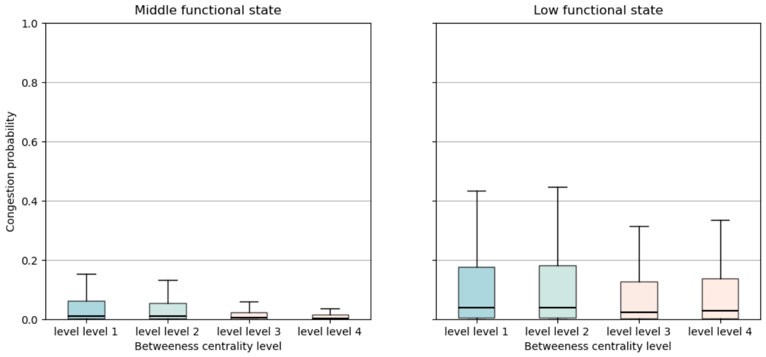

Figure S29. Congestion probability of betweeness centrality levels in Shenzhen, grouped by functional traffic states.

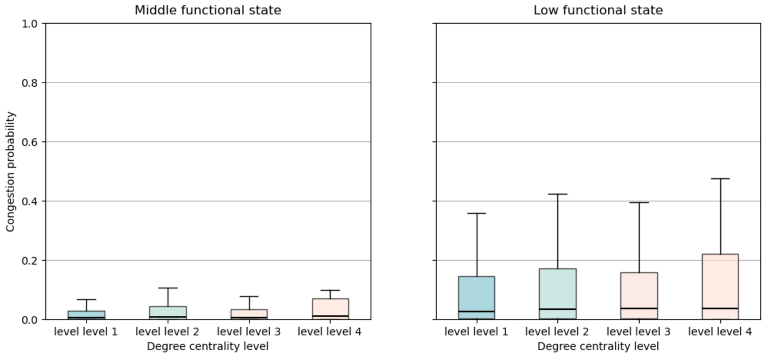

Figure S30. Congestion probability of degree centrality levels in Shenzhen, grouped by functional traffic states.

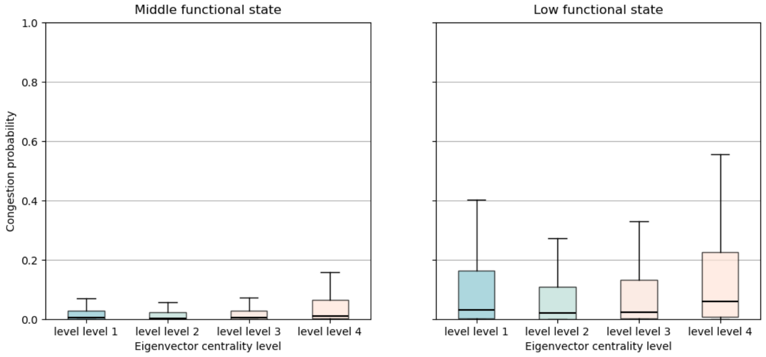

Figure S31. Congestion probability of eigenvector centrality levels in Shenzhen, grouped by functional traffic states.

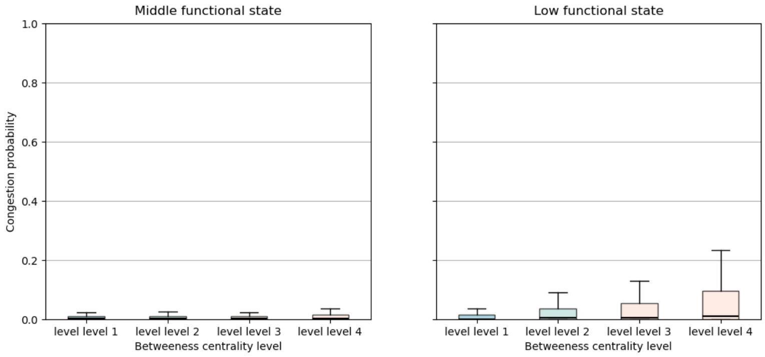

Figure S32. Congestion probability of betweeness centrality levels in Tianjin, grouped by functional traffic states.

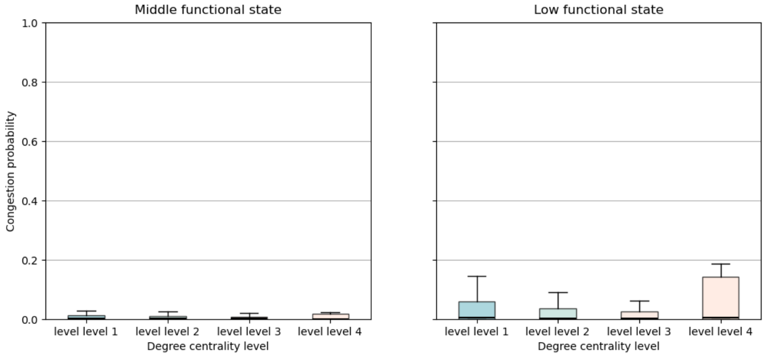

Figure S33. Congestion probability of degree centrality levels in Tianjin, grouped by functional traffic states.

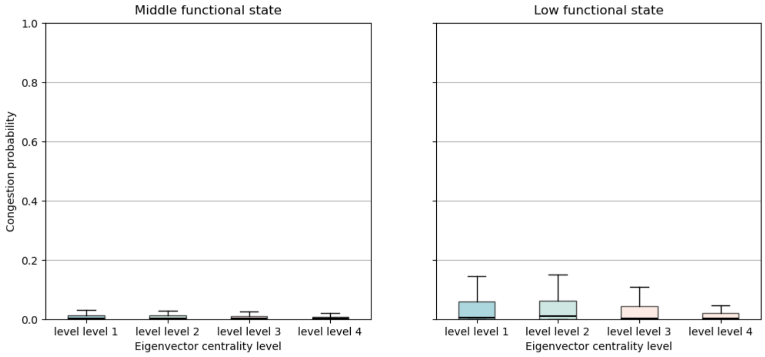

Figure S34. Congestion probability of eigenvector centrality levels in Tianjin, grouped by functional traffic states.

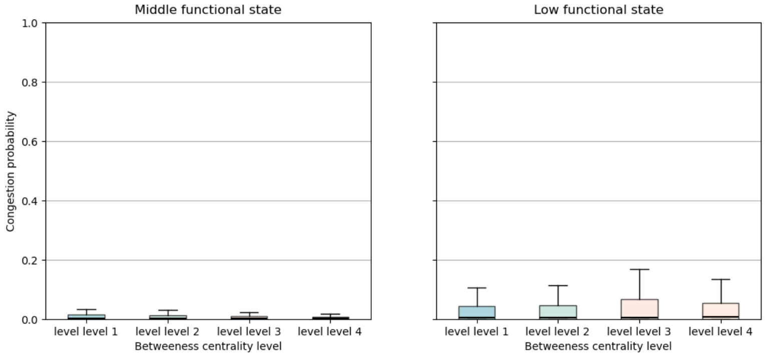

Figure S35. Congestion probability of betweeness centrality levels in Zhengzhou, grouped by functional traffic states.

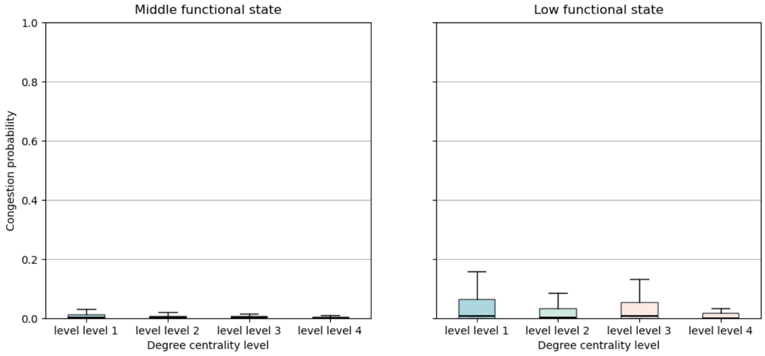

Figure S36. Congestion probability of degree centrality levels in Zhengzhou, grouped by functional traffic states.

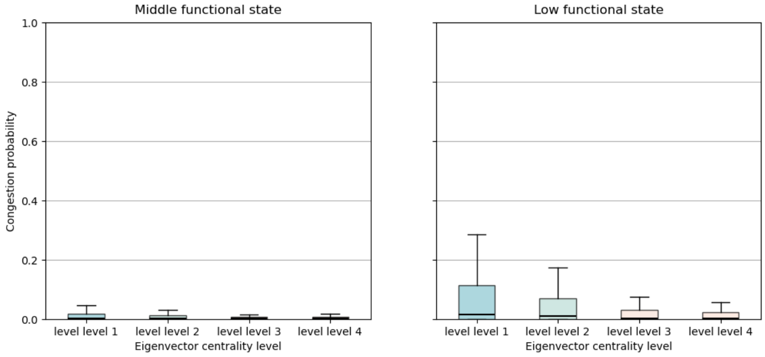

Figure S37. Congestion probability of eigenvector centrality levels in Zhengzhou, grouped by functional traffic states.

4. Statistical Test Results

Table S3. Kruskal–Wallis test results of different levels in a same functional states.

City	State	H-Statistic	*p*-Value
Beijing	High	495.554	0.000
Beijing	Middle	289.679	0.000
Beijing	Low	629.775	0.000
Changsha	High	915.835	0.000
Changsha	Middle	183.283	0.000
Changsha	Low	110.712	0.000
Chengdu	High	279.386	0.000
Chengdu	Middle	90.292	0.000
Chengdu	Low	470.178	0.000

Table S3. *Cont.*

City	State	H-Statistic	*p*-Value
Guangzhou	High	796.031	0.000
Guangzhou	Middle	18.533	0.000
Guangzhou	Low	424.017	0.000
Guiyang	High	372.196	0.000
Guiyang	Middle	173.478	0.000
Guiyang	Low	28.792	0.000
Hangzhou	High	340.589	0.000
Hangzhou	Middle	15.428	0.001
Hangzhou	Low	191.935	0.000
Jinan	High	306.023	0.000
Jinan	Middle	43.222	0.000
Jinan	Low	274.079	0.000
Nanjing	High	288.606	0.000
Nanjing	Middle	29.627	0.000
Nanjing	Low	38.223	0.000
Tianjin	High	430.836	0.000
Tianjin	Middle	34.166	0.000
Tianjin	Low	336.356	0.000
Wuhan	High	181.769	0.000
Wuhan	Middle	130.419	0.000
Wuhan	Low	372.689	0.000
Shanghai	High	391.666	0.000
Shanghai	Middle	134.537	0.000
Shanghai	Low	380.942	0.000
Shenzhen	High	1129.472	0.000
Shenzhen	Middle	852.973	0.000
Shenzhen	Low	93.275	0.000
Zhengzhou	High	386.689	0.000
Zhengzhou	Middle	16.911	0.001
Zhengzhou	Low	84.619	0.000

Table S4. Summary of Mauchly's test of different levels in different functional states.

City	F	*p*-Value	np2
Beijing	40.332883	9102.725564	0
Changsha	15.928209	4809.772161	0.000
Chengdu	33.147065	5551.791527	0.000
Guangzhou	34.886478	6212.876945	0.000
Guiyang	9.828843	2219.406280	0.000
Hangzhou	46.390198	8048.477043	0.000
Jinan	18.114141	3431.585758	0.000
Nanjing	11.413075	3308.684922	0.000
Tianjin	8.281681	2980.857797	0.000
Wuhan	48.925761	7429.747652	0.000
Shanghai	41.984151	9255.524575	0.000
Shenzhen	36.205580	4715.734198	0.000
Zhengzhou	30.740015	5352.497540	0.000
